# Outpatient Parenteral Antimicrobial Therapy in a Tertiary Hospital in France: A Description of Service Models and Costs

**DOI:** 10.3390/antibiotics14100971

**Published:** 2025-09-26

**Authors:** Espérie Burnet, Alicia Le Bras, Guillaume Roucoux, Christian Dupont, Etienne Canouï, Clément Leclaire, Jérémie Zerbit, Pierre Régis Burgel, Clémence Martin, Isabelle Durand-Zaleski, Martin Duracinsky

**Affiliations:** 1 Respiratory Medicine and National Cystic Fibrosis Reference Center, Cochin Hospital, Assistance Publique Hôpitaux de Paris (AP-HP), 75014 Paris, France; 2 ERN-Lung CF Network, 60596 Frankfurt am Main, Germany; 3 Paris Health Economics and Health Services Research Unit (URC Eco IdF), Hôtel-Dieu Hospital, AP-HP, 75004 Paris, France; 4 ECEVE, UMR-S 1123, Paris Cité University, Inserm, 75010 Paris, France; 5 Infectious Diseases Unit, Cochin Hospital, AP-HP, Paris Cité University, 75014 Paris, France; 6 Paris Public Hospital-at-Home (HAD AP-HP), Greater Paris University Hospitals, AP-HP, 75005 Paris, France; 7 Institut Cochin, Université Paris Cité, Inserm U1016, 75014 Paris, France; 8 INSERM UMR 1153 CRESS Research Center in Epidemiology and Statistics, Paris Cité University, 75005 Paris, France; 9 Internal Medicine Unit, Bicêtre Hospital, AP-HP, 94270 Le Kremlin-Bicêtre, France

**Keywords:** outpatient parenteral antimicrobial therapy, OPAT, hospital to home transition, antimicrobial stewardship, care pathways, care planning, home-based treatment

## Abstract

**Background/Objectives:** Outpatient parenteral antimicrobial therapy (OPAT) has been implemented throughout the world for the treatment of most infections. Published studies have focused on OPAT delivery, with limited data on coordination and monitoring practices. **Methods:** A mixed methods study, using an exploratory sequential design, was conducted at a tertiary hospital in Paris, France. Ten semi-structured interviews were conducted with prescribing physicians and professionals involved in OPAT coordination and monitoring. A general inductive approach was used to analyze verbatim data and build a framework for OPAT model characterization. Cost estimates, using a standardized scenario, were applied to each model. **Results:** Five OPAT coordination and monitoring models were identified. All OPATs were administered by visiting nurses in the patient’s home. Referral to an infectious disease physician was not systematic, and three models, with 3 to 50 OPAT episodes/year each, outsourced hospital-to-home coordination and monitoring to external medical service and device providers. Only one OPAT model, with 450 OPATs annually, included a nurse specialist within the unit to coordinate and monitor treatment. Clinically and/or socially vulnerable patients received OPAT through hospital at home services, which reported 30 OPATs/year. Under the standardized clinical scenario applied to each OPAT model, weekly costs ranged from EUR 1445 to EUR 2308. **Conclusions:** The diversity of OPAT coordination and monitoring practices identified within a single hospital suggests that similar trends may be observed in other settings, in France and elsewhere. Identifying the most cost-effective OPAT service model could guide stakeholders and facilitate the implementation of best practice recommendations in line with antimicrobial stewardship principles.

## 1. Introduction

Outpatient parenteral antimicrobial therapy (OPAT) was developed in the 1970s in the United States, initially for the treatment of cystic fibrosis exacerbations in response to increasing healthcare pressures. Since then, the advent of antibiotics with long half-lives, along with the invention of elastomeric pumps and the use of Peripherally Inserted Central Catheters (PICC lines) have paved the way for the outpatient treatment of most infections requiring parenteral antibiotics throughout the world.

OPAT is considered safe and effective [1,2], but the rate of treatment- and catheter-related adverse events (AEs) can reach 30% [2,3] and 33% [1,4] of outpatient episodes, respectively. If the resources available are not sufficient to ensure adequate treatment monitoring and the outpatient management of an AE, hospital readmission or a visit to the Emergency Department (ED) may occur [3]. The effectiveness of OPAT services can therefore be captured in the 30-day hospital readmission rate [5,6], which has been estimated as being between 6% and 26% [1,2,3].

Best practice recommendations [7,8,9] and quality indicators [10,11,12] have been published or updated over the past ten years. Their common objective is not only to ensure patient safety and treatment effectiveness but also to lower the potential for antimicrobial resistance, to which inappropriate prescriptions and treatment interruptions are known to contribute [12]. They emphasize the need for a structured multidisciplinary OPAT team, including an infectious disease (ID) physician, a pharmacist, and a specialist nurse, as well as a standardized follow-up process with seamless communication between the OPAT team and the patient [7,8,10,11].

Various OPAT delivery models have been documented in many countries and can be grouped into four broad categories: outpatient infusion services, home administration by the patient or a caregiver, home administration by a visiting nurse, and hospital-at-home (HaH) services [2,3,13]. A growing body of evidence shows that OPAT team structure and outpatient resources for treatment coordination and monitoring can vary widely within countries [14,15], underscoring a gap in the literature that this study aims to address. In addition, published data on OPAT services and delivery in France remain limited, and the few available studies suggest considerable regional variability [16,17,18].

The main objective of this study was to explore prescription, coordination, and monitoring practices for OPAT prescribed by acute care units in a tertiary hospital in Paris, France, in order to describe existing OPAT service models. A secondary objective was to estimate the costs of each model identified.

## 2. Results

### 2.1. Participant Characteristics

The senior physicians of 14 acute care, ID, and HaH units were contacted by email and/or telephone. Three physicians did not respond (Urology, Nephrology, and Gastro-enterology), and the nurse managers (*n* = 3) of these units did not follow up after initial contact. One physician declined to participate (Cardiology), citing systematic referral to the ID team, and one declined to be recorded (Dermatology). Three units were excluded due to an insufficient number of OPAT prescriptions per year (Ambulatory Surgery, Oncology, and Hematology). Six physicians agreed to participate and were included (one each from Pulmonology, Infectious Diseases, Diabetes, and Orthopedics and two from HaH). One manager (HaH) and three nurses (one in Pulmonology, one discharge facilitator, and one who worked for a Medical Service and Device Provider) were recruited through snowball sampling and agreed to participate.

A total of ten semi-structured interviews were conducted by CD (male, nurse coordinator, 25 years in pulmonology and OPAT), GR (male, sociologist, PhD candidate), and EB (female, advanced practice nurse, 10 years in pulmonology, 3 in OPAT, MSc in Public Health). To reduce potential bias, interviews with pulmonology healthcare professionals were conducted by GR. There were no other pre-existing relationships between researchers and participants. The mean interview duration was 48 min (range: 15 to 122 min).

Five prescribers, one each from Pulmonology, Orthopedics, Diabetes and Endocrinology, Infectious Diseases, and HaH, were interviewed. One Medical Service and Device Provider (MSDP) coordinator, whose background was in nursing and to which the ID, Orthopedics, and Diabetes teams addressed their patients for OPAT, was also interviewed.

Other participants included an additional physician from the HaH unit who did not prescribe OPAT but provided administrative and logistical support, a nurse manager (HaH), a specialist nurse coordinator (Pulmonology), and a hospital-wide discharge facilitator nurse (from an external logistics service provider to which the hospital outsourced hospital to home coordination). Seven of the participants were female, the mean age was 42 years (range: 26 to 55), and experience in OPAT ranged from 3 months to 22 years (Table 1).

The annual number of OPAT courses prescribed and/or coordinated per OPAT service model ranged from three in Orthopedics to 450 in Pulmonology. Acute care units could refer OPAT patients to a mobile unit of three ID physicians, to HaH services, or organize care with an MSDP, which the ID unit also called upon for the hospital to home transition. The same three to five MSDPs worked externally with several acute care units and with the ID team to coordinate and monitor treatment. The antibiotics prescribed included beta-lactams, carbapenems, cephalosporins, and aminoglycosides, either alone or in combination (aminoglycosides with beta-lactams). Treatment duration ranged from 3 days to 6 months.

The Orthopedics unit referred most patients to the ID team or to HaH services, based on clinical severity and social isolation factors, but also managed three to five OPATs per year independently. The Diabetes unit followed 10 OPATs per year for diabetic foot infections, without referral to the ID department, and worked with four MSDPs, including two in common with the ID unit. The Gastroenterology department, also with 10 OPATs per year on average, did not refer patients to the ID unit and coordinated with an external discharge facilitation service, who then contacted an MSDP, or with HaH services for more complex patients. The Pulmonology unit, which followed cystic fibrosis (CF) and non-CF bronchiectasis patients with recurring respiratory infections, had a team of four specialist nurses to coordinate and monitor 450 OPATs per year. Finally, HaH services reported an average of 30 OPATs per year and treated patients addressed by acute care units, with or without initial ID referral, and applied strict eligibility criteria that accounted for clinical severity, dependency, and psycho-social factors.

### 2.2. Overall OPAT Service Model Characteristics

**Figure 1:**
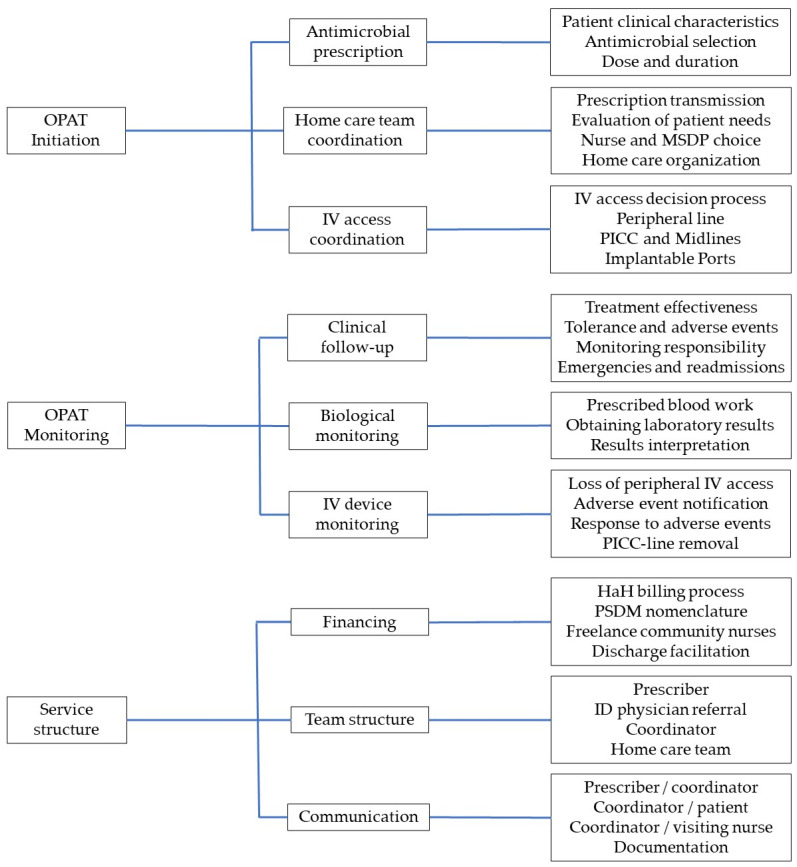
Thematic tree built from interview verbatims using a general inductive approach to describe OPAT service models, showing three broad categories, each including 3 themes, with 4 subthemes per theme. OPAT, outpatient parenteral antimicrobial therapy; MSDP, Medical Service and Device Provider; IV, intravenous; PICC, Peripherally Inserted Central Catheter; HaH, hospital-at-home; ID, Infectious Diseases.

The framework of analysis (thematic tree) summarizing the structural processes and experiences identified in the verbatims included three key themes (initiation, monitoring, and service structure), each containing three sub-themes, and three to four categories per sub-theme (Figure 1). Five OPAT models of coordination and monitoring emerged from these interviews (Table 2).

In all models but the nurse-managed model, OPAT was initiated during a hospital stay. The Pulmonology unit also prescribed OPAT at home without initial hospitalization, after a visit with the attending physician or patient contact with the specialist nurse. None relied on patient or caregiver self-administration or treatment preparation by a pharmacist. All reported referring patients to visiting nurses for antibiotic preparation and administration. Visiting nurses in France are self-employed and are paid for the care they provide through a fee-for-service payment scheme.

The HaH service provided all medications and supplies from its pharmacy and used electronic volumetric pumps for medication administration. In all other models, the antibiotics were supplied by the patient’s local pharmacy and administered exclusively using elastomeric pumps, delivered by an MSDP. For blood work, blood was drawn by visiting nurses and sent to local laboratories, with all participants reporting difficulties in obtaining results, except in the HaH model, which used hospital laboratories in its network and had direct access to results. Venous access devices included short peripheral catheters, PICC lines and Midlines, and implantable ports.

### 2.3. Model Characteristics

#### 2.3.1. Model A: ID Physician Referral and OPAT Coordination by MSDP

In the ID physician referral model, the acute care unit referred the patient to an ID physician who prescribed antibiotics and oversaw follow-up and monitoring thereafter. An initial standardized prescription form was sent to one of three MSDPs. The MSDP contacted a local visiting nurse team, forwarded the prescription to the local pharmacy, and provided the infusion device, preparation kits and IV line dressing kits. The primary contact for the visiting nurse and patient during OPAT was the ID physician, who could be reached during work hours. Treatment-related AEs were not frequent as most occurred before patient discharge and were managed by the ID physician. Catheter-related AEs were referred back to the acute care unit or to the ED. The ID team did not have dedicated hospital beds but collaborated with most acute care units and was responsible for antimicrobial stewardship throughout the hospital. The total cost amounted to EUR 1445.

#### 2.3.2. Model B: Acute Care Unit Prescriber and OPAT Coordination by MSDP

The prescriber was the patient’s attending physician within the acute care unit, who did not systematically seek ID referral. Outpatient care coordination was delegated to an MSDP, following the same process as described in Model A. This model was described in the treatment of diabetic osteomyelitis and bone and joint infections not eligible for HaH. The MSDP coordinated the hospital-to-home transition with the visiting nurse and local pharmacy. It served as an intermediary between patient, nurse, and prescriber for follow-up and monitoring. AEs were managed either in the ED or by the attending physician in the acute care unit. The total costs were identical to those of Model A.

#### 2.3.3. Model C: Nurse-Led OPAT Coordination

In the nurse-led OPAT coordination model, OPAT could be initiated in hospital or in the outpatient setting without initial hospitalization. The majority of patients had bronchiectasis, due to cystic fibrosis or other etiologies, and were often treated for recurring respiratory infections. They were followed by senior physicians who had acquired extensive experience in antimicrobial therapy and prescribed treatment without ID referral. Specialist nurse coordinators were the primary contact for patients, MSDPs, local pharmacists, and visiting nurses. They were specialized in OPAT and IV line management and could provide telephone triage, laboratory results monitoring, and a rapid response to most treatment- and catheter-related AEs, coordinating with the referral physician as needed. The total costs amounted to EUR 1458 (Table 3).

#### 2.3.4. Model D: Discharge Facilitation Nurse and OPAT Coordination by MSDP

In the hospital to home discharge facilitation model, a nurse employed by an external logistics service provider was tasked with collecting prescriptions from the prescribing physician and transmitting them to an MSDP who then managed OPAT implementation and ensured follow-up and monitoring. The discharge facilitator nurse was not physically located in the unit, the MSDP was the primary contact for the patient and visiting nurse and often forwarded any concerns to the prescribing physician without informing the discharge facilitator nurse. The ID team did not participate in OPAT prescription, coordination, or monitoring. The estimated total cost was EUR 1595.

#### 2.3.5. Model E: Hospital at Home

In the HaH model, a discharge coordination nurse from the HaH team assessed the patient for OPAT eligibility before discharge and coordinated care with the HaH pharmacy, physicians, and an HaH liaison nurse who reached out to HaH nursing staff and/or freelance community nurses. Due to staffing shortages within the HaH team, approximately 40% of nursing care was contracted out to freelance community nurses. All medications were provided by the HaH pharmacy, including non-OPAT-related medications. All blood draws were sent to the nearest hospital laboratory within the network, and results could be accessed directly through the patient’s medical file. An HaH physician could be reached by the nursing team 24 h a day, and most treatment-related AEs were managed at home. Catheter-related AEs required readmission to the patient’s referral unit or a visit to the ED. HaH services provided close monitoring of more clinically fragile patients, who would otherwise require hospitalization in an acute care unit, and/or vulnerable individuals with insecure living arrangements or a lack of family support. This model did not involve any MSDPs or local pharmacies. The cost of 7 days of HaH services was estimated to be EUR 2308.

## 3. Discussion

Five different types of OPAT service model were identified within a single tertiary care hospital. All had access to the hospital microbiology laboratory and called on visiting nurses to administer treatment in the patient’s home. ID physician referral was not systematic, but the ID team outsourced hospital to home transition coordination to an MSDP (model A), as did attending physicians without ID referral (model B). Model C included OPAT prescription by a cystic fibrosis physician and outpatient care coordination by specialist nurses who could respond to AEs in the outpatient setting, but no involvement of a clinical pharmacist. More clinically severe and/or socially vulnerable patients were referred to HaH services (model E), which included both continuous monitoring and a clinical pharmacist but did not specialize in OPAT. Model D introduced an additional intermediary between the patient and the prescriber, without systematic ID physician referral or antimicrobial expertise. In all but the HaH model, treatment obtained from the patient’s local pharmacy was prepared in the patient’s home by the visiting nurse using supplies delivered by an MSDP, and delays in obtaining laboratory results were mentioned as an important barrier in the quality of OPAT care. The total costs of the 7-day standardized OPAT scenario ranged from EUR 1444 to EUR 2308.

In recent overviews of OPAT practices around the world, French OPAT delivery models have been described as HaH exclusively [19] or as “ad hoc” [13], a term used to describe a diversity of coordinated care pathways outside of formal OPAT programs and without consistent oversight by an ID specialist. Our study confirms that several OPAT services exist side by side, not only within the country but also, and more importantly, in a single hospital. Two recent studies examining OPAT practices in the UK have also shown wide heterogeneity in service provision, which, the authors suggest, may be due to variability in the availability of specialist expertise [14], a lack of financial resources, a constrained workforce, and/or differences in population density [20]. Similar trends are likely at play in France [16,18,21], and a similar fragmentation of OPAT services may also be observed in other countries.

The structure and processes involved in antimicrobial prescription, hospital to home coordination, and treatment monitoring, in particular, AE identification and response, have been shown to impact OPAT success or failure [22,23,24]. Factors such as inoperative communication channels, delays in obtaining laboratory results, and the lack of outpatient resources to respond to AEs can increase hospital readmissions and ED visits [3,20]. In contrast, the integration of an OPAT nurse to oversee treatment coordination, monitoring, and documentation has been shown to be associated with a reduction in unplanned 30-day readmission rates of approximately 10% [25,26]. International guidelines therefore recommend that OPAT programs ensure a clear distribution of responsibilities and communication channels, and that the team include “a medically qualified clinician (e.g., an infectious diseases physician, internal medicine specialist, cystic fibrosis physician, pediatrician or a surgeon with an infection interest), a medically qualified infection specialist (infectious diseases physician/pediatric infectious diseases specialist or clinical microbiologist), a specialist nurse and a clinical antimicrobial pharmacist” [7].

None of the OPAT service models identified at our hospital fully met these guidelines, as each model failed to include all four key members of the OPAT team. The main barrier to the implementation of a formal OPAT program in line with international recommendations, in our hospital and elsewhere [16,27], has been structural. The limited capacity of the existing ID team to cover AMS needs at the scale of the hospital, including but not limited to OPAT oversight, has so far not received the required administrative and financial support due to competing priorities, despite the demonstrated cost savings associated with avoided prolonged hospital stays [1,28], particularly in high-risk populations [29]. Plans for the creation of an ID unit in the hospital are currently underway and should increase capacity for OPAT oversight throughout the hospital, with the exception of the pulmonology unit, which follows a specific patient population. The integration of a specialist nurse for OPAT coordination and monitoring would enable this expansion to fully meet recommendations for the implementation of a formal OPAT program.

Over the past 10 years, the tasks and responsibilities of nurses specialized in coordination and monitoring have been taken on by MSDPs, whether for OPAT or other treatments administered in the outpatient setting. MSDPs have filled the growing gap in linking hospital prescribers to visiting nurses and local pharmacists and more than 2400 were registered in France in 2018 [30]. They currently lack full recognition as healthcare providers, cannot access a patient’s health-related information, and operate in a highly competitive market [31]. Some, but not all, have specialized in OPAT. The services and the supplies they provide, which our study shows amount to EUR 685 for the first week of treatment, are reimbursed in part by Social Security payments and in part by the patient’s private complementary insurance. To our knowledge, the cost-effectiveness of the service they provide has never been evaluated by an independent body and has not been compared to the coordination of outpatient care by a specialist nurse within the prescribing unit.

Economic analyses of OPAT services have thus far examined the outcomes of various OPAT programs in comparison with inpatient care and included hospital bed-days saved in cost calculations [32,33,34]. In a cost-minimization analysis, Dimitrova and colleagues compared the costs and outcomes of six different OPAT delivery models to those of inpatient care in the UK but assumed that good practice recommendations in terms of OPAT team composition, coordination, and monitoring were systematically followed [33], which is not always verified [14,20]. The design of our study did not allow us to collect outcome data, which prevented any effectiveness analysis, but our findings, along with those of Gilchrist [14] and Durojaiye [20] in the UK, suggest that future economic evaluations of OPAT service models should compare existing services to each other rather than to inpatient care and should focus on coordination and monitoring practices in addition to OPAT delivery.

Our study has limitations that prevent any generalization of our findings. The exploration of OPAT services was limited to a single hospital at a specific point in time. Although participant selection aimed to include diverse viewpoints, some participants had limited OPAT experience, and the prescribing physicians of three of the eight acute care units that were contacted did not participate. It is therefore likely that our findings do not fully represent current OPAT practices in our hospital or in France, although data saturation was reached in the analysis. In addition, cost estimates were based on scenario modeling for one week of treatment rather than prospectively collected data and were limited to outpatient costs; any tasks undertaken by physicians and nurses within the hospital were excluded, as were annual costs. Nonetheless, our findings provide additional insights into existing OPAT services in France.

Given the growing constraints on financial resources in most healthcare systems—regarding which France is no outlier—implementing formal OPAT programs following international guidelines may not be feasible in every healthcare setting. In France, regional disparities in access to physicians, nurses, and tertiary hospitals have been growing over the past 20 years [35], prompting stakeholders to implement healthcare reforms aiming to reduce fragmentation [35]. Our findings allow us to propose a classification of OPAT services into five distinct models, which could be used in further research, in France and beyond, to evaluate the cost-effectiveness of various OPAT service models and determine the minimum requirements for optimal coordination and monitoring.

## 4. Materials and Methods

A mixed methods study was conducted in 2023 at Cochin university hospital (Paris, France) using an exploratory sequential design [36]. In the initial exploratory phase, semi-structured interviews were conducted to describe OPAT coordination and monitoring practices. These findings informed subsequent quantitative cost analysis in the second phase of the study.

Purposive sampling was used to recruit senior physicians in ID and HaH, as well as those practicing in the acute care units most likely to prescribe and/or coordinate OPAT: Cardiology, Dermatology, Orthopedics, Pulmonology, Diabetes and Endocrinology, Nephrology, Urology, and Gastroenterology. Other professionals involved in coordinating and/or monitoring outpatient care, such as nurses, managers, or pharmacists, were recruited through snowball sampling. Eligible participants were included if they were involved in at least three OPAT prescriptions per year, either as a prescriber or as a coordinator. All were informed of the study’s objectives and were asked to provide consent.

Interviews were conducted in the participant’s office or a medical examination room to ensure privacy. A structured interview guide ensured consistency across interviews. It covered the following topics: (1) OPAT initiation (eligibility criteria, physicians involved in treatment prescription, venous access verification); (2) hospital to home transition (composition and structure of the home care team, coordination between the prescriber and the home care team); (3) patient follow-up and monitoring (monitoring for treatment- and catheter-related AEs, communication between the home care team and the prescriber, evaluation of treatment effectiveness); and (4) structural characteristics of the OPAT service.

All interviews were conducted in French, recorded, and transcribed verbatim. All personal identifiers were removed from the transcriptions. Data saturation was considered to have been achieved once all aspects of service coordination, monitoring, and delivery had been addressed. Participants were asked to confirm the accuracy of the description of the OPAT service model pertaining to their practice. No additional interviews were required.

A General Inductive Approach was used to analyze the data [37]. A codebook was developed using an inductive and iterative method, defining new themes and subthemes as analysis of the transcripts progressed. Two researchers (EB and GR) independently analyzed five transcripts and compared codebooks. Any discrepancies in code definition and attribution were discussed and arbitrated, when necessary, by CD or ALB. The remaining transcripts were analyzed by EB, following the agreed-upon code definitions. Further inductive categorization into themes and greater categories was undertaken by EB and CD. The interview transcripts were analyzed using NVivo 14 software (QSR International, Doncaster, Australia).

Outpatient care costs were estimated from a healthcare system perspective for each identified OPAT service model. All resources involved in treatment coordination and administration in the outpatient setting were identified, regardless of their nature or funding sources, from the initiation of the hospital to home transition until treatment discontinuation. Costs were calculated based on a standardized 7-day treatment of 12 g Piperacillin-Tazobactam in 240 mL of normal saline once daily, administered as a continuous infusion through a PICC line, requiring one blood draw to monitor treatment tolerance, and PICC line removal upon treatment discontinuation. Costs included hospital to home transition fees, nurse visits, medications and normal saline, supplies and equipment, one laboratory test during the 7-day period, transportation to the clinic for PICC line removal, and home hospitalization days for the HaH model. Unit costs for each resource were drawn from the national health insurance reference pricing schemes [38,39,40] and are detailed in Appendix A Table A1 and Table A2. Ethical approval was obtained from the Inserm institutional review board (IRB00003888, ref: 23-994).

## 5. Conclusions

Five different OPAT coordination and monitoring models were identified in a single tertiary hospital in France. Although OPAT was delivered exclusively by visiting nurses in the patient’s home, the intervention of an ID physician, of an antimicrobial pharmacist, or of a specialist nurse were not systematic, and the resources available to coordinate and monitor treatment in the outpatient setting varied widely from one OPAT service model to the next, which may impact unplanned readmission rates. As financial constraints on healthcare systems grow, access to quality OPAT services may become more unequal. In light of antimicrobial stewardship recommendations and current disparities in access to healthcare services, further research into the effectiveness and cost-effectiveness of existing OPAT coordination and monitoring practices in France and elsewhere would help in better understanding the factors associated with OPAT service quality, success, and failure.

## Figures and Tables

**Table 1:** Participant characteristics (n = 10).

Unit/Department	Profession	Gender	Age (Years)	Experience in Unit	Roles in OPAT Service
Orthopedics	Physician	Female	40	6 years	Prescription
Pulmonology	Physician	Female	52	22 years	Prescription
Pulmonology	Specialist Nurse	Female	54	3 months	Coordination Monitoring Telephone triage
Infectious Diseases	Physician	Male	37	5 years	Prescription Monitoring
Diabetes	Physician	Female	34	3 years	Prescription
HaH	Manager	Female	55	11 years	Coordination Team management
HaH	Physician	Male	35	2 years	Prescription
HaH	Physician	Male	41	6 years	Prescription Monitoring
Hospital discharge facilitation	Nurse	Female	26	4 months	Coordination Monitoring
MSDP	Nurse	Female	46	4 years	Coordination Monitoring

OPAT, outpatient parenteral antimicrobial therapy; MSDP, Medical Service and Device Provider; HaH, hospital-at-home.

**Table 2:** OPAT service model characteristics.

Model	OPAT Initiation	OPAT Monitoring	OPAT Service Structure
A 50 per year	Prescription: In acute care unit, by ID physician Discharge coordination: MSDP IV access coordination: By attending physician or ID physician prior to discharge	Clinical follow-up: Evaluation of treatment effectiveness and tolerance by ID physician. Response to AE in outpatient visit, in ED, or on readmission Biological monitoring: Patient discharged after tolerance verified. Local laboratory if required; results difficult to obtain IV device monitoring: By visiting nurse. Response to AE in ED or acute care unit. PICC line removal by ID physician or in acute care unit as outpatient	Financing: MSDP coordination fee and supplies, local pharmacy, freelance visiting nurse Team structure: Two ID physicians. Coordination externalized to MSDP Communication: ID physician can be reached (phone, email) by patient, visiting nurse, and MSDP during working hours Documentation: By ID physician, in electronic medical record accessible by acute care referral physician
B 3 to 10 per year	Prescription: By attending physician in acute care unit; ID referral not systematic Discharge coordination: MSDP IV access coordination: By attending physician prior to discharge	Clinical follow-up: Evaluation of treatment effectiveness by MSDP and during next scheduled visit with attending physician. Tolerance evaluated by MSDP. Response to AE in outpatient visit, in ED, or on readmission Biological monitoring: Patient discharged after tolerance verified. Local laboratory if required; results difficult to obtain IV device monitoring: By visiting nurse. Response to AE in ED or acute care unit. PICC line removal in acute care unit as outpatient	Financing: MSDP coordination fee and supplies, local pharmacy, freelance visiting nurse Team structure: Attending physicians (variable); coordination externalized to MSDP Communication: Attending physician can be reached (phone, email) by MSDP during working hours. MSDP relays nurse and patient concerns Documentation: In hospital electronic medical record on discharge and during next patient visit
C 450 per year	Prescription: By attending physician with expertise in antimicrobial therapy; no ID referral Discharge coordination: Specialist nurse in acute care unit IV access coordination: By specialist nurse prior to discharge or as outpatient	Clinical follow-up: Evaluation of treatment tolerance and effectiveness by specialist nurse during first week and at end of OPAT, by specialist nurse or during next scheduled visit with attending physician. Response to AE and PICC line removal in acute care unit as outpatient Biological monitoring: Patient discharged after tolerance verified. Local laboratory if required; results obtained by specialist nurse IV device monitoring: By visiting nurse. Response to AE and PICC line removal in unit as outpatient by specialist nurse	Financing: Specialist nurse coordination fee, MSDP coordination fee and supplies, local pharmacy, freelance visiting nurse. Outpatient PICC line removal fee Team structure: Five attending physicians, four specialist nurses Communication: Specialist nurses can be reached (phone, email) by patient and visiting nurse Documentation: In hospital electronic medical record throughout OPAT
D 10 per year	Prescription: By attending physician in acute care unit; no ID referral Discharge coordination: Discharge facilitator nurse and MSDP IV access coordination: By attending physician prior to discharge	Clinical follow-up: Evaluation of treatment effectiveness during next scheduled visit with attending physician. Tolerance evaluated by MSDP and discharge facilitator (redundancy). Response to severe AE in ED or on readmission Biological monitoring: Patient discharged after tolerance verified. Local laboratory if required; results difficult to obtain IV device monitoring: By visiting nurse. Response to AE in ED or acute care unit PICC line removal in acute care unit as outpatient	Financing: Discharge facilitation fee, MSDP coordination fee and supplies, local pharmacy, freelance visiting nurse Team structure: Attending physicians (variable); coordination externalized to discharge facilitator Communication: Attending physician can be reached (phone, email) by MSDP during working hours. MSDP relays nurse and patient concerns, often without notifying discharge facilitator Documentation: In hospital electronic medical record on discharge and during next patient visit
E 30 per year	Prescription: By attending physician in acute care unit, with or without ID referral Discharge coordination: HaH discharge coordinator and liaison nurse IV access coordination: By attending physician prior to discharge or HaH physician	Clinical follow-up: Daily evaluation of treatment tolerance and effectiveness by HaH nursing team and physician. Response to AE through HaH services, in ED, or on readmission if severe Biological monitoring: As needed, through nearest laboratory within network IV device monitoring: By visiting nurse. Response to AE in ED or acute care unit. PICC line removal by HaH nurse	Financing: Patient Diagnosis Group baseline fee, adjusted for dependency score and treatment time, including all antimicrobials, equipment, and supplies Team structure: HaH physicians on call 24 h a day. HaH nurse coordinators are distributed throughout hospital network and liaison nurses and HaH nurses are distributed by geographical sector; 40% of care is contracted out to freelance community nurses Communication: HaH physician can be reached by phone 24/7 Documentation: In electronic medical record specific to HaH—not accessible to acute care unit

OPAT, outpatient parenteral antimicrobial therapy; ID, Infectious Diseases; MSDP, Medical Service and Device Provider; AE, adverse event; ED, Emergency Department; PICC, Peripherally Inserted Central Catheter; HaH, hospital-at-home.

**Table 3:** Outpatient costs, in EUR, for 7-day standardized OPAT services.

Services Billed to National Health Insurance	MSDP (Models A and B)	Nurse-Led (Model C)	Discharge Facilitation (Model D)	Hospital at Home (Model E)
Medical service and device provider				
Home infusion one-time set-up fee	229	229	229	-
Weekly monitoring	46	46	46	-
Weekly devices and supplies	410	410	410	-
Pharmacy	226	226	226	-
Visiting nurse	369	369	369	-
Local laboratory blood test processing	18	18	18	-
Hospital-based outpatient nursing care				
OPAT coordination	-	13	-	-
PICC line removal	13	13	13	-
Patient transportation for PICC line removal	134 *	134 *	134 *	-
HaH, first week	-	-	-	2308 †
Hospital-based discharge facilitation	-	-	150 ‡	-
**Total**	**1445**	**1458**	**1595**	**2308**

OPAT, outpatient parenteral antimicrobial therapy; MSDP, Medical Service and Device Provider; HaH, hospital-at-home; PICC, Peripherally Inserted Central Catheter. Source unless otherwise specified: National Health Insurance reference pricing payment scheme. All costs in EUR, calculated based on a standardized weekly scenario including Piperacillin-Tazobactam 12 g daily in 240 mL normal saline administered by continuous infusion via elastomeric pump through a PICC line, one blood draw including complete blood count, electrolytes, renal and liver function tests, and C-reactive protein, and outpatient in-hospital PICC line removal. * Average amount submitted for reimbursement in 2024; † diagnosis-related group costs in the public hospital setting; ‡ internal document.

## Data Availability

The data presented in this study (verbatims in French) are available on request from the corresponding author to ensure participant confidentiality.

## Abbreviations

The following abbreviations are used in this manuscript:

OPAT	Outpatient Parenteral Antimicrobial Therapy
ID	Infectious Diseases
ED	Emergency Department
AE	Adverse Event
MSDP	Medical Service and Device Provider
HaH	Hospital at Home
PICC	Peripherally Inserter Central Catheter

## Appendix A

**Table A1:** Nursing services billed by freelance community nurses for one week of outpatient parenteral antimicrobial therapy.

	D0	D1	D2	D3	D4	D5	D6	D7
Patient evaluation and care planning	1							
Infusion preparation, initiation, and 12 h monitoring		1	1	1	1	1	1	1
PICC line dressing change				1				
Blood draw				1				
Flat rate travel allowance		1	1	1	1	1	1	1
Weekend surcharge								1

D, day; PICC, Peripherally inserted central catheter.

**Table A2:** Line item costs used to calculate the total weekly costs of each OPAT model.

Billable Services	Unit Costs (EUR)	Units Per Week (n)	Total Weekly Costs (EUR)
Medical service and device provider			
Home infusion one-time coordination fee	229.0	1	229.0
Weekly monitoring	45.8	1	45.8
Weekly equipment and supplies	409.5	1	409.5
Pharmacy, mean (min-max)			
Piperacillin-tazobactam, 4 g	9.75	21	204.75
Normal saline, 240 mL daily	3.03	7	21.21
Visiting nurse			
Patient evaluation and care planning	12.6	1	12.6
Infusion preparation and initiation	44.1	7	308.7
PICC line dressing change	14.8	1	14.8
Blood draw	4.7	1	4.7
Flat rate travel allowance	2.8	7	19.6
Weekend surcharge	8.2	1	8.2
Local laboratory blood test processing			
Complete blood count	5.2	1	5.2
Serum electrolytes	2.6	1	2.6
Serum creatinine	1.6	1	1.6
Complete liver panel	6.5	1	6.5
C-reactive protein	2.1	1	2.1
Hospital-based outpatient nursing care			
OPAT coordination	12.6	1	12.6
PICC line removal	13.2	1	13.2
Patient transportation for PICC line removal	67.0 *	2	134.0
HaH, DRG daily rate during first week	329.7 †	7	2307.9
Hospital-based discharge facilitation	150.0 ‡	1	150.0

OPAT, outpatient parenteral antimicrobial therapy; PICC, Peripherally Inserted Central Catheter; HaH, hospital at home; DRG, diagnosis-related group. Source unless otherwise specified: National Health Insurance reference pricing payment scheme. * Average amount submitted for reimbursement in 2024. † Diagnosis-related group costs in the public hospital setting. ‡ Internal document.
